# Thermographic Evaluation of the Hands of Pig Slaughterhouse Workers Exposed to Cold Temperatures

**DOI:** 10.3390/ijerph14080838

**Published:** 2017-07-26

**Authors:** Adriana Seára Tirloni, Diogo Cunha dos Reis, Eliane Ramos, Antônio Renato Pereira Moro

**Affiliations:** 1 Tecnological Center, Federal University of Santa Catarina, Florianópolis 88040-370, Santa Catarina, Brazil; diogo.biomecanica@gmail.com (D.C.d.R.); eliane.ramos43@gmail.com (E.R.); renatomoro60@gmail.com (A.R.P.M.); 2 Biomechanic’s Laboratory, CDS, Federal University of Santa Catarina, Florianópolis 88040-90, Santa Catarina, Brazil

**Keywords:** slaughterhouse, thermal sensation, workers, hands, cold

## Abstract

Brazil was rated the fourth leading producer and exporter of pork meat in the world. The aim of this study was to evaluate the temperature of the hands of pig slaughterhouse workers and its relation to the thermal sensation of the hands and the use of a cutting tool. The study included 106 workers in a pig slaughterhouse. An infrared camera FlirThermaCAM E320 (Flir Systems, Wilsonville, OR, USA) was used to collect the images of the dorsal and palmar surfaces of both hands. A numerical scale was used to obtain the thermal sensation. Chi-square test, Pearson correlation and Student’s *t* test or Wilcoxon were used (*p* ≤ 0.05). The majority of workers felt cold in the hands (66%) and workers who used the knife felt the coldest. There was an association between the thermal sensation and the use of knife (*p* = 0.001). Workers who used the tool showed correlation between the thermal sensation and the temperatures of the left fingers, with a difference between the temperatures of the right and left hands of those who used the knife (*p* ≤ 0.05). The hands (left) that manipulated the products presented the lowest temperatures. Findings indicate that employers of pig slaughterhouses should provide gloves with adequate thermal insulation to preserve the health of workers’ hands.

## 1. Introduction

In 2015, Brazil was rated the fourth leading producer and exporter of pork meat in the world [1]. Vergara and Pansera [2] analyzed the activity of boning shoulder in a pig slaughterhouse in the city of Ipiranga-SC/Brazil. The findings suggested that employees were exposed to ergonomic risk related to the physical environment and physical load, and presented high incidence of pain in the wrist, shoulder and lower back. According to Kyeremateng-Amoah et al. [3], workers in pork meatpacking have high rates of acute injuries and chronic disease. 

There are several risk factors for the incidence of upper limb work-related musculoskeletal disorders (UL-WMSDs) in slaughterhouse workers: repetitive work [4,5,6,7,8], artificially cold environments [6,9,10], use of manual tools, and consequently, the application of force in the tasks [6,8,11,12,13]; and the use of gloves [6,14]. 

Occupational exposure to cold may occur through contact with cold air, immersion in water, or by touching cold surfaces, involving the whole body or only a body region [15]. Prolonged exposure to cold often associated with insufficient clothing or physical activity may result in whole-body cooling and a decrease in core temperature. This is further aggravated by exposure to wind or cold water, which increases heat loss through convection [15]. 

Blood flow to the extremities decreases rapidly upon exposure to cold, in order to maintain the body’s core temperature for optimal function of vital organs. Due to vasoconstriction in peripheral microvasculature regions and a high surface/volume ratio, the skin temperature of the fingers tends to rapidly and exponentially decrease to a level approaching that of the environment [16]. Beyond immediate impairment, continued cold exposure and vasoconstriction can also lead to non-freezing cold injuries from reduced nutritional blood flow leading to necrosis [17]. 

The study of temperature using thermal imaging has widespread applications across science and industry. It is used to analyze inflammatory arthritis, osteoarthritis, soft tissue rheumatism as muscle spasm and injury, enthesiopathies, tennis elbow, fibromyalgia, complex regional pain syndrome, peripheral circulation, malignant diseases and other applications [18]. 

Thermographic imaging has been used for analysis of hand structures that are subjected to controlled effort [19]; the thermoregulation after immersion in cold water [20]; to examine the skin temperature of the hand after short task as an indicator of upper extremity musculoskeletal disorder severity [21,22,23,24]; and association of the hands temperatures with use of cutting tool (knife) in a poultry slaughterhouse in Brazil [25]. 

The need for such study is justified by the lack of research analyzing the temperatures of the hands of pig slaughterhouse workers. It is important to point out that there are differences between the production processes of swine and poultry meat. In poultry slaughterhouses the pace is faster (12,000 birds per hour) than in pig slaughterhouses (900 pigs per hour) [26]. Currently, the highest levels of mechanization are found in poultry slaughterhouses; however, in slaughterhouses that kill larger animals, many operations are still carried out manually [26]. These differences may influence skin temperature. According to Havenith [27], the heat loss is proportional to the skin surface area available for heat exchange. 

Studies in diverse meat slaughterhouses are needed in order to identify possible differences and provide information that guides health and safety professionals in adopting measures, to promote the health of these workers. Therefore, the aim of this study was to evaluate the temperature of the hands and its relation with the thermal sensation of the hands and the use of a cutting tool (knife) in pig slaughterhouse workers. 

## 2. Materials and Methods 

The Ethics Committee of the Federal University of Santa Catarina, Brazil (2098/11), in accordance with the Helsinki Declaration, approved this study. It was conducted in a pig slaughterhouse in the South of Brazil, with about 2000 workers. The participants worked in artificially cold environment (8 °C to 12 °C), and were provided with personal protective equipment with Certificate of Approval (CA) by the Brazilian Ministry of Labor (clothes, aprons, gloves, socks and boots).

The daily working time was 8 h and 48 min, which included 15 min for snack, 45 min for meal, two periods of 8 min for physiological necessities and 8 min of worksite physical exercise.

### 2.1. Select of Participants

The workers were randomly selected from a list of names of the processing pig meat sector that included two shifts of work. The requirement for participation in the study was that the worker would have to be performing the same activity in the workstation for at least 15 min prior to data collection, following the recommendations proposed by ISO 11079: 2007 for the calculation of required clothing insulation (IREQ) [28]. In order to eliminate the possibility of skin temperature alterations, the following exclusion criteria were adopted: do not smoke [29], do not be sleep deprived prior the assessment [30], no alcoholic beverage 12 h preceding data collection [31], and no females during menstruation [32].

### 2.2. Participants

The study included 106 workers, 59 females and 47 males with a mean age of 31.0 years (range 18 to 51 years) and 36.7 years (range 19 to 59 years), respectively. The majority of the workers was right-handed (94.3%) and used a knife (61.5%) during the activity prior to data collection. The workers were employed in this sector for at least one month and maximum of 34 years (average 8.5 ± 7.9 years).

### 2.3. Instruments

**Figure 1:**
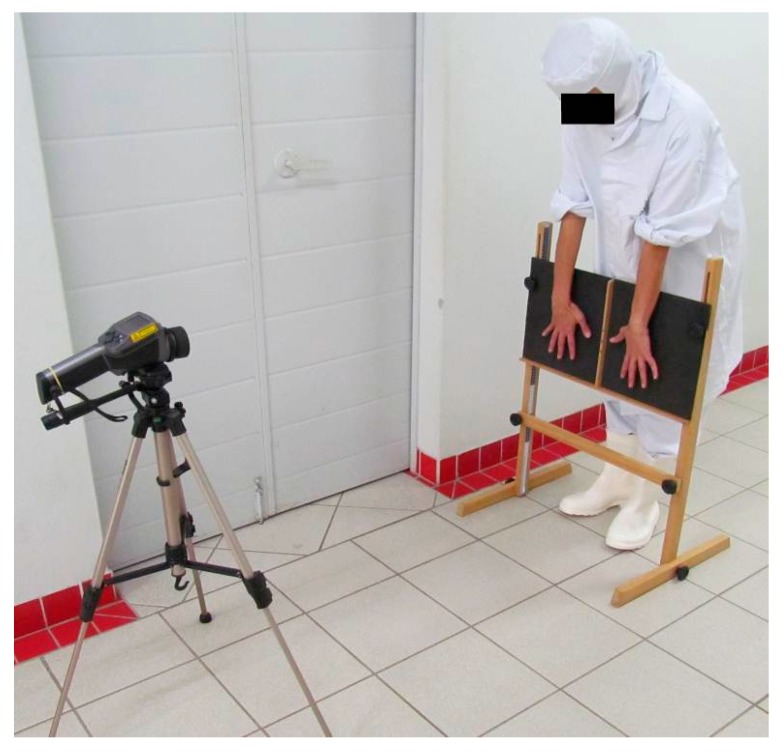
Infrared portable camera and frame for capturing the thermographic images.

In order to record the two thermographic images (dorsum and palm of the hands), an infrared portable camera ThermaCAM^®^ E320 (Flir Systems, Wilsonville, OR, USA) was used. The camera was positioned 1.2 m away from the participant and 0.88 m above the floor (Figure 1. Infrared portable camera and frame for capturing the thermographic images.), and the temperature (≈23 °C) and humidity (≈50%) of the room were recorded for analysis of the images, adopting a 0.98 emissivity (body human). A frame was designed for the current study to capture the thermographic images (Figure 1).

A numerical scale was used to analyze the thermal sensation of the hands, where zero indicated feeling neutral, +1, +2, and +3 indicated feeling slightly warm to feeling very hot, and −1, −2, and −3 indicated feeling slightly cold to very cold [33].

### 2.4. Procedures

Data collection took place in a room located next to the workstations. The time between leaving the workstations and recording the thermographic images was not greater than three minutes. The worker was instructed not to touch any objects and not join hands until the end of recording the images. Upon arrival in the room, the worker firstly removed the gloves and quickly positioned hands for capture the thermographic images (Figure 1).

**Figure 2:**
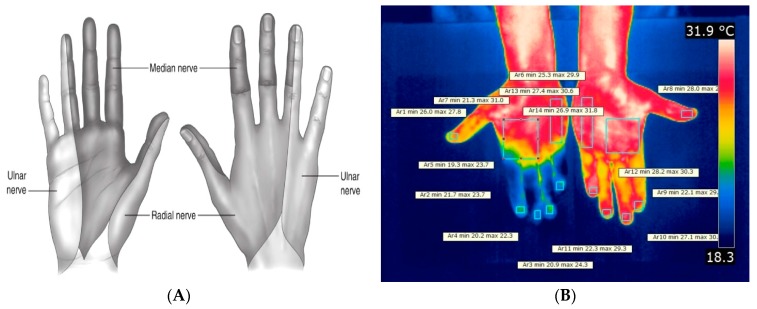
(**A**) Sensory mapping model of the peripheral cutaneous innervation of the hand on the dorsal and palmar surfaces. The figure shows the innervations for the ulnar nerve, radial nerve, and median nerve [34]; (**B**) Thermographic image of the palms of the hands, with the anatomical regions delineated showing the corresponding temperatures for each area.

The areas of peripheral cutaneous innervation distribution in the hand, according to Dincer and Samut [34] (Figure 2), were used to determine the body regions analyzed in this study. The following nomenclature was adopted for each finger analyzed: finger 1 (thumb), finger 2 (index), finger 3 (middle), finger 4 (ring), finger 5 (little). 

Two thermographic images were collected, one of the dorsal surface of the hands and one of the palmar surface (Figure 2B). The regions selected to analyses were the finger’s extremities and approximately two-thirds of the distal aspect of the dorsum of the hands related to the radial innervation, and at least one-half of the ulnar innervation on the medial surface of the dorsum. In relation to the palm, similar regions were selected according to the ulnar and median innervations (Figure 2A). 

After capturing the thermographic images, each worker was instructed to point to a number in the thermal sensation numerical scale, which represented their perception of thermal sensation of the hands. Only one numerical value was recorded for both hands.

### 2.5. Data Analysis

The temperature difference between the left and right hand was calculated considering the predetermined regions, and the criteria adopted for acceptable limits of thermal asymmetry between hands (>1 °C and >0.3 °C), proposed by Hong et al. [35] and Brioschi et al. [36].

The classification used as a reference is described in the ISO 11079:2007 norm—“Ergonomics of the thermal environment”, which recommends frequent temperature monitoring of the fingers in the workplace, and suggests that these temperatures should be above 24 °C for the preservation and proper functioning of the hands [28]. This standard norm states that temperatures up to 15 °C are considered high physiological stress, characterized by peripheral vasoconstriction, irregular perspiration, and a thermal sensation of cold. 

The Flir QuickReport software version 1.2 SP2 2009 (Flir Systems, Wilsonville, OR, USA) was used, as shown in Figure 2B. The software provided the minimum, maximum and average temperatures of the selected body regions. 

### 2.6. Statistical Analysis

Regarding to statistical analysis, the workers were divided into two distinct groups: those who used the knife (58) and those who did not use (48). In order to compare the average or median temperatures of the fingers, dorsal and palm regions of the right with the left hand between the groups, firstly, normality of data was tested, and depending on the results, the Student’s t test or the Wilcoxon test was performed, both of dependent samples.

The Chi-square test was used to compare the thermal sensation of the hands among workers who used the cutting tool and those who did not use; and the difference in temperature between the left and right hands, considering the acceptable limits of thermal asymmetry between hands (>1 °C and >0.3 °C); and lastly, to compare the classification of the average temperatures (≤24 °C and >24 °C) between the right and left hands of the two groups. 

The Pearson correlation was used to correlate the temperature of the regions of the hands with the scale of the thermal sensation reported by two groups of workers. A value of *p* ≤ 0.05 was considered to be significant for all tests.

## 3. Results

The results revealed that 66% of workers (n = 70) felt cold in the hands at different intensities: (−1) 25.5%, (−2) 16.0% and (−3) 24.5%. There was association between the use of knife and the thermal sensation reported by the participants (*p* = 0.001). The majority of these workers who used the knife (81%) felt cold in their hands.

The group that did not used the knife had significant positive correlations between thermal sensation of the hands and the temperature of the right fingers (r = 0.29−0.38; *p* = 0.046–0.009), and left fingers (r = 0.29–0.31; *p* = 0.045–0.033) on the dorsal surface; the same for the palmar surface of the right fingers; (r = 0.29–0.38; *p* = 0.05–0.009), except the right thumb on the dorsal surface (r = 27; *p* = 0.66). However, for workers who used the knife, significant positive correlations was found only in the fingers of the left hand, both on the dorsal (r = 0.28–0.35; *p* = 0.036–0.007) and palmar surface (r = 0.28–0.35; *p* = 0.036–0.008). 

The comparison between the temperatures of the hands (right and left) for the two groups is shown in Table 1. No significant difference was found between the mean temperatures of the fingers, palmar and dorsal regions, as well as between left and right side in workers who did not use the knife. The workers who used the tool presented significantly lower mean and median values for the left hands.

The difference between average temperatures of the right and left hands, frequency and percentage of cases using the criterion (ΔT >1 °C or >0.3 °C) for the two groups are shown in Table 2.

The majority of workers showed temperature differences (>0.3 °C) between each region analyzed when comparing between the right and left hands in both groups. However, only in the group that used the knife, the majority of workers presented a classification of >1 °C for both the dorsum and the palm of the hands. There was a significant difference (*p* ≤ 0.05) on the number of workers that presented difference of temperature of >1 °C between hands (right and left) for the following regions: fingers 1, 3, 4, 5 and ulnar and radial regions of the dorsum of hand; fingers 1, 3, 4 and ulnar and median regions of the palm of hand when using the knife. 

The temperatures of right and left hand fingers, according to the recommendations of ISO 11079:2007 (≤15 °C, 15.1 °C to 24 °C and >24 °C), are shown in Table 3.

In relation to the mean of the temperatures classified as ≤24 °C and >24 °C, there was significant statistical differences between right and left hands only for the group of workers that used the knife: the fingers 3 (*p* = 0.048) and 4 (*p* = 0.026) in the dorsal surface, and in the fingers 2 (*p* = 0.028) and 5 (*p* = 0.021) in the palmar surface of the hands (Table 3). These results showed that the majority of the workers who use the knife presented temperatures of ≤24 °C in the left hands, suggesting that it was due to the handling of cold product with the contralateral hand (non-dominant).

## 4. Discussion

Studies indicate that the majority of workers at a poultry slaughterhouse, who perform their activities in artificially cold environments, felt cold 54.1% [9] and 78% felt cold in the hands [25]. No research that analyzed the thermal sensation of pig slaughterhouse workers was found. 

A study on the temperature of the hands in a poultry slaughterhouse in Brazil, with 227 workers, using the same numerical scale as in present study, analyzed the thermal sensation of the hands utilizing thermographic images. The results showed that there was no significant association (*p* = 0.937) between the use of a tool (knife) and the perception of cold [25]. It is suggested that the results of Ramos et al. [25] were different from the present study due to the difference in size of product handled (poultry slaughterhouse), versus the present study (pigs), which may indicate that these workers had more direct contact with the products through the hands. Holmér et al. [15] states that occupational exposure to cold may occur due to some factors, such as contact with cold surfaces. Considering that, the heat loss is proportional to the skin surface area available for heat exchange [27]. 

Ramos et al. [25] found significant correlation between the thermal sensation of the hands and the temperatures of the fingers (dorsal and palmar surface of the fingers of both hands) for the workers of a poultry slaughterhouse who did not use a knife, with the exception of the dorsum and palm of the hands (*p* ≤ 0.05). These results are also not compatible with the present study, which found no correlation between temperatures of the palmar surface of the left fingers and the dorsal surface of the fingers of both hands (*p* > 0.05), for this same group. 

The findings of the present study may be associated with the fact that most workers were right-handed and handle the products with their dominant side (right). Concerning the limitation of this study, when inquiring about the thermal sensation, the questions of perception of cold were not specific for each hand, and the workers may have reported the most predominant thermal sensation that was felt. 

When analyzing the group who used the knife, Ramos et al. [25] found that there was no correlation between the temperatures of all regions of the hands and the thermal sensation reported by the workers. Unlike the current study, where there was significant positive correlation for the fingers of the left hand for both palmar and dorsal surfaces. In pig slaughterhouses, the size of the manipulated product and the duration of the task cycles are greater than in poultry meat processing companies [26]. These greater numbers contribute to longer periods of contact with frozen products, while the opposite hand manipulates the cutting tool. 

The first sign of cold exposure is the sensation of cold, followed by cardiovascular, metabolic, and endocrine responses [15]. Therefore, it is important to study the relation of the thermal sensation with the temperature of the hands, given the difficulty of companies having a thermal camera available to monitor these temperatures.

Ramos et al. [25] found that there was no significant difference between the temperatures of the fingers, dorsum and palm regions of the hand (left and right) in workers who did not use the knife for both surfaces of the hands, corroborating with the present study. However, the majority of workers who used the cutting tool showed lower temperature values on the left hand for fingers 1 and 2 (*p* < 0.001), and for fingers 3, 4, and 5 (*p* < 0.009), with no significant difference only for the ulnar and radial regions of the dorsal surface and ulnar regions of the palm of the left hands [25]. By contrast, in our study, there was significant difference for all regions. Again, these discrepancies may also be related to the larger size of meat products, consequently increasing fingers/hand exposure to cold. 

Cummings [10] analyzed 300 injury incident reports from a pork processing plant in Nebraska, and, of the 91 hand/wrist/forearm injuries, 57 (63%) occurred in cold environments, in which the main source of injury was the tasks that required the use of handheld tools. Also, of the 108 finger injuries (majority of the body parts injured), 81 (75%) occurred in the cold worksite with 15 (18.5%) resulting from exposure to handheld tools. Tirloni et al. [9] investigated 290 workers of a poultry slaughterhouse and found that there was an association between body discomfort and perception of cold (*p* = 0.035).

Similar results were found in Ramos et al. [25], where the majority of the workers of the poultry slaughterhouse showed a temperature difference of >0.3 °C between the regions analyzed for the right and left hands in both groups. In addition, the majority of the tool users presented temperature differences of >1 °C for both the dorsum and the palm of the hands; and the proportion of workers with differences of >1 °C was significantly higher in the group that used the knife. 

Imray et al. [17] suggests that in case of frostbite of tissues, rapid re-warming of a non-freezing cold injury is advised. In Brazil, the Standard Regulatory Norm 36 (NR-36) [37] establishes requirements for activities performed at meat processing industries. The norm requires that workers should have a hand warming system available near toilets or break rooms, when manual activities are performed in cold environments or require constant contact with cold surfaces and cold products. 

The ISO 11079 recommends that finger temperatures should be higher than 24 °C during prolonged exposure, or down to 15 °C when sporadic [38]. Ramos et al. [25] found significant statistical differences between the right and left hand for the group of workers using the knife, the temperatures <24 °C were more frequent for fingers 1, 2, 3 and 4 in the dorsal and palmar surface of the hands. Conversely, the present study showed significant difference for the same group of workers, however only for the fingers 3 and 5 (dorsal surface) and fingers 2 and 5 (palmar surface) presented more frequency of temperatures <24 °C. 

In Ramos et al. [25] the difference between temperatures of the hands of workers who used the tool may have occurred due to the handling cold product with the contralateral side. The present study corroborates with Ramos et al. [25] and adds other factors that may exert interference on the hand’s temperature: the hand that holds the knife applies force during the cutting task [39], wearing gloves increases muscle activity [26,40]. Finally, there was no difference in the temperatures of the left and right hands of workers who did not used the knife, which could be justified by the manipulation of products with both hands. 

According to Vogel et al. [13], meat cutters face higher risks of injury and musculoskeletal problems than most other occupational groups. The data from the company explored in this study showed a positive trend for the reduction of injuries and sick leave, by implementing changes such as reducing the time using the cutting tool to a maximum of 6 h per day, and introducing a job rotation scheme with work periods of equal length. 

Kyeremateng-Amoah et al. [3] investigated the incidence of worker injuries, lacerations, and infections reported by 10 companies from 2004 to 2009 and found that the average annual mean total injury rates were 6.4 per 100 workers (poultry) and 13.2 per 100 workers (pork). In addition, in pig slaughterhouses, the sharp tools were the largest source of lacerations (65.4%), while products were the largest source of skin infections (55.8%). Claudon and Marsot [12] states that work performed with sharp knives reduces the biomechanical stress in the upper limbs (UL); however, for Kyeremateng-Amoah et al. [3], the use of sharp tools and set line speeds can cause injuries, lacerations, and infections. 

Furthermore, aside from the use of tools, Buckle [14] points out that the cold can directly influence the incidence of UL-WMSDs and through indirect means, requiring the use of protective equipment (gloves). According to Tappin et al. [41], wearing gloves may act as a risk factor to increase the magnitude or duration of force applied during hand-gripping in order to cut the product. For Buckle [14], the muscles must exert greater force to overcome the restrictions of wearing gloves, and as a result, increases the difficulty in coordinating and applying the appropriate force. 

According to Reis et al. [8], manual grip strength may be a predictor of upper limb compression syndrome due to peripheral nerve compression, contributing to loss of grip strength. Moreover, Oska [42] suggests that decrease in body temperature results in increased muscular effort, which can contribute to the development of musculoskeletal disorders. 

In addition, Tirloni et al. [9] recommends to employers of a poultry slaughterhouse the use of very sharp knives to reduce the effort required to perform cutting tasks through proper training of knife honing/sharpening operators; the use of adequate clothing according to the environment’s temperature and to the handled products, respecting the biological individuality of workers, promoting thermal insulation and comfort. Occupational Safety and Health Administration [6] recommends employers to train employees of poultry slaughterhouses on proper care, use, handling techniques and sharpening of knives and scissors; and use of any special tools and devices, as well as use of safety equipment, including personal protective equipment (PPE), to prevent WMSDs, and proper fitting of gloves. 

The initial response to whole-body or local exposure of the extremities to cold is a strong vasoconstriction, leading to a rapid decrease in hand and foot temperature. This impairs tactile sensitivity, manual dexterity and muscle contractile characteristics while increasing pain and sympathetic drive, decreasing gross motor function, occupational performance, and survival [16]. Therefore, working in the cold environments, handling cold products and using handheld tools and gloves should be monitored, and measures taken to prevent WMSDs.

## 5. Conclusions

The majority of workers felt cold in the hands at different intensities. There was association between using the knife and the thermal sensation reported by the participants, were those who used the knife felt most cold in the hands. In the group of workers who did not use the knife there was a significant positive correlation in most fingers of the right hand, both on the dorsal and palmar surfaces. Conversely, for the knife users, there was correlation only with the fingers of the left hands, in both surfaces (dorsal and palmar). In the comparison between hand temperatures, the temperature did not present a significant difference between workers who did not use the knife, as opposed to the group that used the knife, since the hand’s temperature values were lower in the non-dominant hands, which handled cold products.

Most workers who use the knife presented differences of temperature between the right and left hands (<1 °C), with lower temperatures for the left hands. There was significant differences between right and left hand medium temperatures classified as ≤24 °C and >24 °C, only for workers who used the knife, with left hands presenting values ≤24 °C.

The results of this study suggest that the employers of pig slaughterhouses should provide gloves with adequate thermal insulation according to the temperature of the products handled by the workers, avoiding the overlap of gloves, which compromises the tactile sensitivity and dexterity of the hands; replace protective equipment regularly in order to maintain its thermal insulation capacity; instruct of workers on the risks of working and handling cold products; perform job rotation with tasks that do not require the use of handheld tools and in environments with higher temperatures; and, finally, monitor workers’ upper extremity health conditions, since these areas are exposed to risk factors that can trigger work-related musculoskeletal disorders.

## Figures and Tables

**Table 1:** Presentation of the temperature values (°C) of the analyzed body regions and the comparison between the mean or median temperature of the hands (right and left) as the use of the knife by workers.

**Without Knife**								
**Dorsal Surface**	**Innervation**	**Minimum**		**Maximum**		**Mean/Median**		***p*** **#**
		**R**	**L**	**R**	**L**	**R**	**L**	
Finger 1	Median	22.3 ± 5.1	22.3 ± 5.4	24.9 ± 5.9	24.6 ± 6.0	26.6	25.3	0.652
Finger 2	Median	21.6 ± 5.4	21.6 ± 5.3	23.4 ± 6.0	23.6 ± 6.0	23.1	24.5	0.916
Finger 3	Median	21.7 ± 5.6	21.6 ± 5.3	23.3 ± 6.1	23.7 ± 6.0	23.0	24.8	0.447
Finger 4	Median/ulnar	21.8 ± 5.7	21.5 ± 5.3	23.5 ± 6.2	23.7 ± 6.0	24.8	25.3	0.209
Finger 5	Ulnar	21.4 ± 5.5	21.0 ± 5.2	23.0 ± 6.2	23.5 ± 5.9	23.9	25.0	0.560
Ulnar region	Ulnar	22.7 ± 3.8	23.0 ± 4.0	26.8 ± 3.1	27.0 ± 3.5	25.6	25.5	0.200
Median region	Median	22.1 ± 3.8	22.6 ± 4.0	28.6 ± 2.4	28.8 ± 2.6	25.9	26.6	0.142
**Palmar Surface**								
Finger 1	Median	22.3 ± 5.0	22.2 ± 4.9	23.7 ± 5.2	23.4 ± 5.1	25.1	24.8	0.266
Finger 2	Median	21.5 ± 4.9	21.2 ± 4.8	22.7 ± 5.2	22.8 ± 5.2	22.7	23.1	0.564
Finger 3	Median	21.5 ± 4.9	21.6 ± 4.9	22.6 ± 5.0	22.9 ± 5.0	22.6	23.4	0.512
Finger 4	Median/ulnar	21.3 ± 5.0	21.5 ± 4.8	22.6 ± 5.2	22.7 ± 5.1	23.5	23.0	0.702
Finger 5	Ulnar	21.1 ± 5.0	21.4 ± 5.0	22.2 ± 5.3	22.8 ± 5.1	22.9	23.4	0.505
Ulnar region	Ulnar	23.5 ± 4.0	23.8 ± 4.2	27.5 ± 3.6	27.5 ± 3.8	26.5	27.0	0.739
Median region	Median	23.1 ± 4.1	23.2 ± 4.3	30.0 ± 2.3	30.1 ± 2.3	27.7	27.6	0.793
**With Knife**								
**Dorsal Surface**	**Innervation**	**Minimum**		**Maximum**		**Mean/Median**		***p*** **#**
		**R**	**L**	**R**	**L**	**R**	**L**	
Finger 1	Radial	22.6 ± 4.8	21.1 ± 4.6	25.0 ± 5.6	23.3 ± 5.2	22.4	21.7	<0.001 *
Finger 2	Median	21.3 ± 5.3	19.8 ± 4.6	22.8 ± 5.9	21.8 ± 5.2	19.6	19.3	0.009 *
Finger 3	Median	21.2 ± 5.3	19.3 ± 5.0	22.8 ± 5.8	21.1 ± 5.3	20.1	19.5	<0.001 *
Finger 4	Median/ulnar	21.0 ± 5.4	19.3 ± 5.1	22.6 ± 6.0	21.2 ± 5.5	19.9	19.4	0.002 *
Finger 5	Ulnar	21.0 ± 5.1	18.5 ± 4.5	22.3 ± 5.8	20.3 ± 5.1	19.4	17.4	<0.001 *
Ulnar region	Ulnar	23.2 ± 3.5	21.2 ± 3.4	28.7 ± 2.3	27.8 ± 2.7	26.1 ± 2.8	24.9 ± 3.0	0.001 *
Radial region	Radial	22.8 ± 3.4	20.8 ± 3.1	30.5 ± 1.7	29.6 ± 1.8	27.3 ± 2.3	26.1 ± 2.2	<0.001 *
**Palmar Surface**								
Finger 1	Median	22.7 ± 4.8	21.1 ± 4.3	24.1 ± 5.1	22.8 ± 4.6	22.1	21.1	<0.001 *
Finger 2	Median	21.5 ± 4.8	20.5 ± 4.3	22.7 ± 5.1	21.9 ± 4.5	20.2	20.2	0.021 *
Finger 3	Median	21.3 ± 4.8	19.9 ± 4.3	22.6 ± 5.0	21.2 ± 4.4	21.3	19.6	0.001 *
Finger 4	Median/ulnar	21.1 ± 4.8	20.0 ± 4.3	22.3 ± 5.2	21.3 ± 4.5	20.1	19.2	0.035 *
Finger 5	Ulnar	21.0 ± 4.9	19.4 ± 4.4	22.1 ± 5.2	20.6 ± 4.6	19.7	18.7	0.001 *
Ulnar region	Ulnar	24.9 ± 3.7	22.8 ± 3.8	29.6 ± 2.8	28.3 ± 3.3	27.7 ± 3.1	26.2 ± 3.4	<0.001 *
Median region	Median	24.5 ± 3.9	21.8 ± 3.5	31.7 ± 1.9	31.0 ± 1.9	28.6 ± 2.7	27.2 ± 2.6	<0.001 *

# *p*-Value refers to comparison of the average temperatures of the body regions between the right and left hands; Wilcoxon test—presented median; Student’s *t* test—mean and standard deviation; * *p* < 0.05; R—right; L—left.

**Table 2:** Differences between temperature averages of the right and left hands of the two groups.

	Without Knife (n = 48)					With Knife (n = 58)				
Regions of the Hand	Dorsal Surface									
	ΔT	ΔT > 1 °C ^†^		ΔT > 0.3 °C ^‡^		ΔT	ΔT > 1 °C ^†^		ΔT > 0.3 °C ^‡^	
	$\bar{X}$ ± s	n	%	n	%	$\bar{X}$ ± s	n	%	n	%
Finger 1	1.1 ± 1.1	22	45.8	39	81.3	3.0 ± 2.7	46	79.3	54	93.1
Finger 2	1.6 ± 1.6	24	50.0	39	81.3	2.5 ± 2.6	37	63.8	54	93.1
Finger 3	1.4 ± 2.0	19	39.6	40	83.3	3.1 ± 3.0	43	74.1	55	94.8
Finger 4	1.3 ± 1.8	19	39.6	36	75.0	3.0 ± 2.9	39	67.2	50	86.2
Finger 5	1.5 ± 2.0	19	39.6	37	77.1	3.2 ± 3.4	37	63.8	54	93.1
Ulnar region	1.0 ± 1.2	17	35.4	36	75.0	2.1 ± 1.9	39	67.2	53	91.4
Radial region	1.0 ± 0.9	17	35.4	36	75.0	1.8 ± 1.5	34	58.6	54	93.1
Total	1.3 ± 1.6	137	40.8	263	78.3	2.7 ± 2.7	275	67.7	374	92.1
	**Palmar Surface**									
Finger 1	1.1 ± 0.9	22	45.8	38	79.2	2.7 ± 2.5	38	65.5	53	91.4
Finger 2	1.5 ± 1.5	26	54.2	39	81.3	2.2 ± 2.4	35	60.3	48	82.8
Finger 3	1.2 ± 1.7	19	39.6	39	81.3	2.6 ± 2.5	37	63.8	56	96.6
Finger 4	1.3 ± 1.9	19	39.6	38	79.2	2.7 ± 2.4	36	62.1	55	94.8
Finger 5	1.5 ± 1.9	21	43.8	43	89.6	2.6 ± 3.0	35	60.3	52	89.7
Ulnar region	1.0 ± 0.9	15	31.3	41	85.4	2.2 ± 2.2	39	67.2	48	82.8
Median region	0.9 ± 0.8	14	29.2	40	83.3	1.9 ± 1.7	37	63.8	51	87.9
Total	1.2 ± 1.4	136	40.5	278	82.7	2.4 ± 2.4	257	63.3	363	89.4

∆T—Difference between temperatures averages of the hands (right and left); ^†^ Criterion of 1.0 °C established by Hong et al. [35]; ^‡^ Criterion of 0.3 °C established by Brioschi et al. [36].

**Table 3:** Classification and comparison between right and left fingers temperatures, according to ISO 11079:2007 [28].

**Without Knife (n = 48)**							
	**Right Hand n (%)**			**Left Hand n (%)**			
**Dorsal Surface**	**≤15 °C**	**15.1−24 °C**	**>24 °C**	**≤15 °C**	**15.1−24 °C**	**>24 °C**	***p*** **#**
Finger 1	2 (4.2)	19 (39.6)	27 (56.2)	2 (4.2)	20 (41.7)	26 (54.1)	0.837
Finger 2	3 (6.3)	22 (45.8)	23 (47.9)	3 (6.3)	20 (41.7)	25 (52.1)	0.683
Finger 3	2 (4.2)	23 (47.9)	23 (47.9)	4 (8.3)	18 (37.5)	26 (54.2)	0.414
Finger 4	3 (6.3)	21 (43.8)	24 (50.0)	4 (8.3)	18 (37.5)	26 (54.2)	0.838
Finger 5	5 (10.4)	20 (41.7)	23 (47.9)	2 (4.2)	21 (43.8)	25 (52.1)	0.838
Total	15 (6.3)	105 (43.8)	120 (50.0)	15 (6.3)	97 (40.4)	128 (53.3)	
**Palmar Surface**							
Finger 1	2 (4.2)	20 (41.7)	26 (54.2)	2 (4.2)	20 (41.7)	26 (54.2)	1.000
Finger 2	4 (8.3)	22 (45.8)	22 (45.8)	3 (6.3)	25 (52.1)	20 (41.7)	0.538
Finger 3	2 (4.2)	24 (50.0)	22 (45.8)	2 (4.2)	25 (52.1)	21 (43.8)	0.837
Finger 4	2 (4.2)	25 (52.1)	21 (43.8)	2 (4.2)	24 (50.0)	22 (45.8)	0.837
Finger 5	4 (8.3)	22 (45.8)	22 (45.8)	4 (8.3)	23 (47.9)	21 (43.8)	1.000
Total	14 (5.8)	113 (47.1)	113 (47.1)	13 (5.4)	117 (48.8)	110 (45.8)	
**With Knife (n = 58)**							
	**Right Hand n (%)**			**Left Hand n (%)**			
**Dorsal Surface**	**≤15 °C**	**15.1–24 °C**	**>24 °C**	**≤15 °C**	**15.1−24 °C**	**>24 °C**	***p*#**
Finger 1	1 (1.7)	30 (51.7)	27 (46.6)	2 (3.4)	36 (62.1)	20 (34.5)	0.186
Finger 2	4 (6.9)	30 (51.7)	24 (41.4)	4 (6.9)	36 (62.1)	18 (31.0)	0.246
Finger 3	4 (6.9)	30 (51.7)	24 (41.4)	11 (19.0)	34 (58.6)	13 (22.4)	0.048 *
Finger 4	3 (5.2)	32 (55.2)	23 (39.7)	11 (19.0)	33 (56.9)	14 (24.1)	0.114
Finger 5	2 (3.4)	33 (56.9)	23 (39.7)	10 (17.2)	37 (63.8)	11 (19.0)	0.026 *
Total	14 (4.8)	155 (53.4)	121 (41.7)	38 (13.1)	176 (60.7)	76 (26.2)	
**Palmar Surface**							
Finger 1	1 (1.7)	30 (51.7)	27 (46.6)	1 (1.7)	39 (67.2)	18 (31.0)	0.086
Finger 2	1 (1.7)	33 (56.9)	24 (41.4)	3 (5.2)	42 (72.4)	13 (22.4)	0.028 *
Finger 3	1 (1.7)	36 (62.1)	21 (36.2)	4 (6.9)	42 (72.4)	12 (20.7)	0.064
Finger 4	2 (3.4)	35 (60.3)	21 (36.2)	3 (5.2)	43 (74.1)	12 (20.7)	0.064
Finger 5	1 (1.7)	36 (62.1)	21 (36.2)	7 (12.1)	42 (72.4)	9 (15.5)	0.021 *
Total	6 (2.1)	170 (58.6)	114 (39.3)	18 (6.2)	208 (71.7)	64 (22.1)	

^#^ Comparisons between right and left hands’ medium temperatures classified as ≤24 °C end > 24 °C; Chi-Square test; * *p* < 0.05.
